# A Prototype VP-PET Imaging System Based on Highly Pixelated CdZnTe Detectors

**DOI:** 10.3390/s20051294

**Published:** 2020-02-27

**Authors:** Yongzhi Yin, Yingguo Li, Tianguan Wang, Chuan Huang, Zhenqian Ye, Gongping Li

**Affiliations:** 1 School of Nuclear Science and Technology, Lanzhou University, Gansu 730000, China; liyg17@lzu.edu.cn (Y.L.); wangtq17@lzu.edu.cn (T.W.); huangch18@lzu.edu.cn (C.H.); yezhq13@lzu.edu.cn (Z.Y.); ligp@lzu.edu.cn (G.L.); 2 Engineering Research Center for Neutron Application Technology, Ministry of Education, Lanzhou University, Lanzhou 730000, China

**Keywords:** cadmium zinc telluride (CdZnTe) detectors, imaging applications, Monte Carlo simulation, positron emission tomography (PET)

## Abstract

We investigated a prototype virtual-pinhole positron emission tomography (PET) system for small-animal imaging applications. The PET detector modules were made up of 1.3 mm lutetium-yttrium oxyorthosilicate (LYSO) arrays, and the insert detectors consisted of 0.6 mm pixelated cadmium zinc telluride (CdZnTe). To validate the imaging experiment, we did a Monte Carlo simulation for the virtual-pinhole PET (VP-PET) system in the Geant4 Application for Emission Tomography (GATE). For a point source of ^22^Na with a 0.5 mm diameter, the filtered back-projection algorithm-reconstructed PET image showed a resolution of 0.7 mm full-width-at-half-maximum. The system sensitivity was 0.46 cps/kBq at the center of the field view of the PET system with a source activity of 0.925 MBq and an energy window of 350 to 650 keV. A rod source phantom and a Derenzo phantom with ^18^F were also simulated to investigate the PET imaging ability. GATE simulation indicated that sources with 0.5 mm diameter could be clearly detected using 0.6 mm pixelated CdZnTe detectors as insert devices in a VP-PET system.

## 1. Introduction

Dedicated positron emission tomography (PET) scanners for small-animal studies have been investigated frequently in the past decades. The imaging resolution of a state-of-the-art small-animal PET scanner is usually limited to approximately 1 mm full-width-at-half-maximum (FWHM), which is not enough to carry out quantitative studies of a mouse brain. The targeted volume of an entire mouse brain is as small as 5 mm^3^. Thus, a PET imaging system with sub-0.5 mm spatial resolution is required to acquire complex mouse brain images. Extensive efforts have focused on improving detectors’ intrinsic spatial resolution using such devices as a scintillation detector, a semiconductor detector, and a gas detector. To improve the image resolution of conventional PET scanners, some novel PET geometries have also been proposed. One such PET system is called a virtual-pinhole PET (VP-PET) [1,2,3,4,5,6,7], which uses high-resolution detectors integrated into a commercial PET scanner to achieve both high resolution and high sensitivity.

Cadmium zinc telluride (CdZnTe) detectors are proposed as high-resolution imaging detector candidates because of their room temperature operation, good spatial resolution, high energy resolution, and relatively high detection efficiency for gamma rays [8,9,10,11,12]. Monte Carlo (MC) simulation offers a cost-effective and useful method to understand the imaging ability of a PET based on a pixelated CdZnTe detector [13]. Many studies have reported aspects of both simulations and experiments for prototype conventional PET systems using CdZnTe detectors [14,15]. However, few studies have focused on simulating a PET insert system [16]. One of the challenges is the coincidence detection of two gamma-ray annihilation photons from an insert detector and a PET scanner detector. What remains unresolved is that it is difficult to simulate the behavior of two photons generated from one prompt electron-positron annihilation in two kinds of detectors simultaneously. Researchers need to define the insert detector and the PET scanner detector simultaneously in the MC simulation to track the two photons and calculate the energy deposition in both detectors.

The Geant4 application for emission tomography (GATE) is a widely used simulation software application for emission tomography [17]. GATE has been used to validate many PET imaging systems for both animal studies and human clinics, including the ECAT EXACT HR+ [18], ECAT HRRT [19], Hi-Rez [20], Allegro [21], GE Advance [22], MicroPET Focus 220 [23], Inveon PET/SPECT/CT [24], Mosaic [25], and Biograph mMR [26]. At the time of writing, there have been no published papers in which GATE software was used to validate a PET insert system. GATE v7.0 and later versions can simulate triple-coincidence between two detection systems in one MC run. Researchers can define insert detectors integrated into the PET scanner and analyze the photon hits in both the PET scanner detector and the insert detector. In this paper, to build a VP-PET system, we define lutetium-yttrium oxyorthosilicate (LYSO) detector as PET scanner detectors and CdZnTe detector as insert detectors in GATE.

## 2. Materials and Methods

### 2.1. Small-Animal PET Configurations and Monte Carlo Simulations

**Figure 1:**
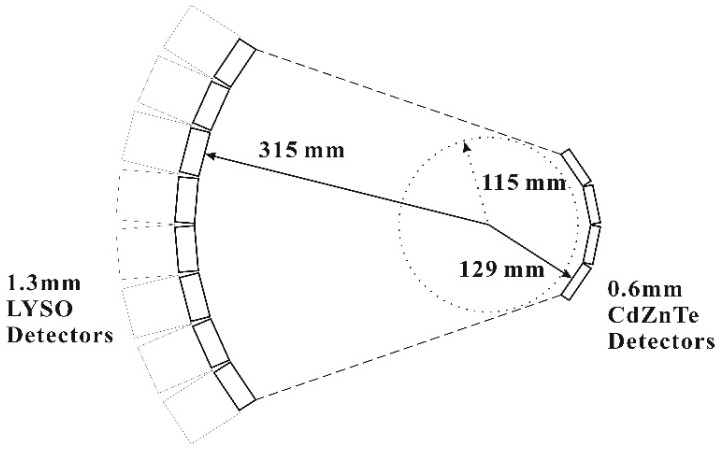
Experimental setup of the prototype positron emission tomography (PET) imaging system. The insert detectors were 0.6 mm pixelated CdZnTe detectors, and the PET scanner detectors were lutetium-yttrium oxyorthosilicate (LYSO) arrays with a 1.6 mm pitch.

The proposed small-animal PET system is shown in Figure 1. Two partial rings are included. The outer ring indicates PET scanner detectors, which comprise of eight LYSO detector modules. Each LYSO detector is arranged in an 18 × 18 crystal array. Every crystal element is molded into a square cross-section of 1.2 mm × 1.2 mm with a 10 mm length. The gaps between elements are 0.1 mm wide. The inner ring serves as insert detectors and has four CdZnTe detector modules. Each CdZnTe detector consists of 16 × 16 pixelated elements. The pitch of a CdZnTe detector is 0.6 mm, and the thickness is 5 mm. In this simulation, we did not mimic the behavior of charge sharing of the CdZnTe detector. Therefore, we selected only the single-pixel photopeak events of pixelated CdZnTe detectors from the individual CdZnTe pixels.

The trajectory radii of the LYSO and CdZnTe detectors were 315 mm and 129 mm, respectively. The two detector modules were arranged into an asymmetric geometry. With the virtual-pinhole PET geometry, the projection of radioactivity distribution on the surface of the LYSO detector was magnified 3.5-fold. The image resolution of this system can be estimated by the equation of $R_{img}=1.25\sqrt{R_{src}^{2}+R_{180}^{2}+R_{det}^{2}+BE^{2}}$ [1]. Where, 1.25 is the factor induced by the image reconstruction. $R_{src}$ is the effective source dimension. $R_{180}$ is the acolinearity effect. $R_{det}$ is the intrinsic resolution of the detector system. BE is the block effect. By calculations, the intrinsic spatial resolution of this prototype PET scanner was approximately 0.426 mm FWHM, and the system resolution was 0.956 mm FWHM.

**Figure 2:**
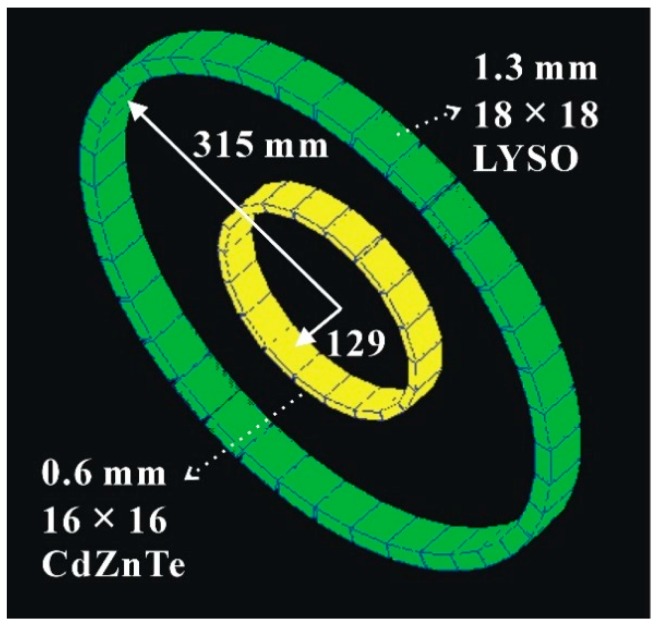
Geant4 application for emission tomography (GATE) simulation geometry of the prototype PET imaging system. The LYSO detectors and CdZnTe detectors were divided into two full rings to acquire coincidence events.

To simulate the performance of the prototype PET imaging system, we defined the PET system by arranging LYSO detectors and CdZnTe detectors into two entire rings, as shown in Figure 2. By this design, we were able to acquire three kinds of coincidence data. Those were coincidence events between the LYSO detectors in the PET scanner ring [scanner–scanner (SS) events], coincidence events between CdZnTe detectors in the insert ring [insert–insert (II) events], and coincidence events between the PET scanner detectors and the insert detectors (IS events). The coincidence data of the II and SS events were recorded directly in GATE output files, whereas the IS coincidence data were calculated offline using the data sets of single file outputs from GATE [6].

The physics processes of the GATE simulation included photoelectric absorption, Compton scatter, Rayleigh scatter, electron ionization, bremsstrahlung, and multiple scattering. In this MC simulation, the PET scanner model is based on the detector modules used in the laboratory measurement. We set 20% energy resolution for LYSO and 10% energy resolution for CdZnTe. An energy window of 350 to 650 keV was defined. We set a coincidence window of 20 ns and a time offset of 500 ns, due to the long electron drift time of CdZnTe detectors that we measured in the actual experiments [7]. The electron trapping and hole trapping were not simulated. We reconstructed the three coincidence data sets using a Fan Beam filtered back-projection (FBP) algorithm [27]. The positron source was ^18^F with 10 kBq radioactivity. We also defined a rod source phantom and a Derenzo phantom in GATE simulation.

### 2.2. Spatial Resolution and System Sensitivity

To obtain the spatial resolution of this small-animal PET system, we imaged point sources throughout the region of interest. For the entire system size, the effective detection range was set from 0 to 95 mm in a transaxial offset. The 0 mm indicates the CFOV of the PET system. The spatial resolution of the system was measured in the radial offsets. A ^22^Na point source in a 0.25 mm diameter with 10 kBq radioactivity was stepped across the insert ring of the PET system. The radial offsets of point sources selected in the MC simulation ranged from 0 to 42 mm due to the limitations of the field of view of the prototype PET scanner. For a 0 to 30 mm radial offset, we moved the point source by 2 mm steps. For a 30 to 42 mm radial offset, we moved the source by 4 mm steps, as shown in the horizontal coordinate of Figure 2. In total, 19 source locations were simulated and recorded by GATE. Coincidence events from the single-pixel photopeak events of the pixelated CdZnTe detector were recorded, and the coincidence events from charge-sharing events of the pixelated CdZnTe detector were rejected.

For a full-ring VP-PET system, the total system sensitivity would be higher than the system sensitivity of conventional PET scanners because of the integration of insert detectors. In this study, a ^22^Na point source was used to calculate the system sensitivity of the current prototype PET system. The radioactivity of the test point source was set to 0.925 MBq, and the energy window was 350 to 650 keV. With the limitations of the GATE software, we could record only coincidence events from the single-pixel photopeak events of the pixelated CdZnTe detector.

### 2.3. Source Phantom PET Imaging

The imaging performance of the PET system was characterized using point sources and phantoms filled with ^18^F. The point source of ^18^F was a 0.5 mm diameter sphere, and the activity was 10 kBq. A Derenzo phantom with ^18^F sources was also defined. The sources had diameters ranging from 0.5 to 2.0 mm. We calculated the detecting matrix and reconstructed it using the FBP algorithm. The dimension of the detecting matrix hinged on the number of detector elements. In the x–y plane, the insert ring had 1344 elements, and the PET scanner ring had 1512 elements. In the GATE simulation, 2 × 10^5^ events were tracked. For source imaging data acquisition, we defined the positron source as a back-to-back gamma-ray mode. We chose back-to-back gamma-ray sources to speed up the MC simulation. By doing so, the positron range and acolinearity effects were not included in the MC study.

## 3. Results

### 3.1. Spatial Resolution

**Figure 3:**
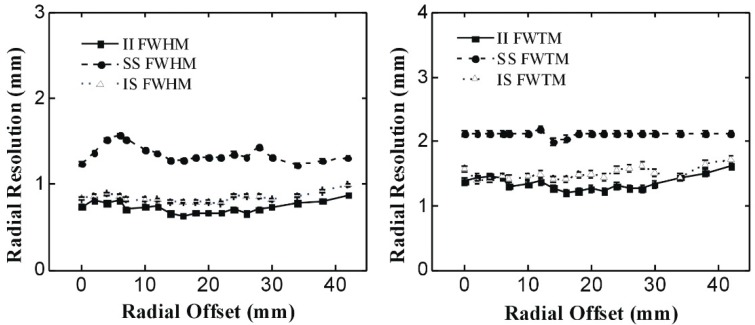
Radial resolution distribution through the PET system in the radial offsets; both full-width-at-half-maximum (FWHM)(left) and full-width-at-tenth-maximum (FWTM) (right) are shown.

Figure 3 shows the radial spatial resolution of the PET system as the point source shifted along the radial direction. Both FWHM and full width at tenth-maximum (FWTM) resolutions are shown in Figure 3. The radial resolution decreases when the source shifts to the edge of the PET system. It was confirmed that the radial resolution was affected by the depth of interaction of detection events from the PET system. The average FWHM of radial resolution obtained with the II, IS, and SS coincidence events in the CFOV of the PET system were 0.74 mm, 0.83 mm, and 1.24 mm respectively. The standard errors of radial resolution FWHM are less than ± 0.02 mm across the system. The FWTM of radial resolution obtained with the II, IS, and SS coincidence data in the CFOV of the PET system were 1.38, 1.58, and 2.13 mm. The standard errors of radial resolution FWTM are less than ± 0.05 mm across the system. In the entire range of the insert ring, the radial resolutions of the PET system reconstructed by the II, IS, and SS coincidence data at FWHM were 0.63 to 0.87 mm, 0.78 to 0.99 mm, and 1.22 to 1.57 mm respectively, and the radial resolutions obtained by the II, IS, and SS coincidence data at FWTM were 1.20 to 1.61 mm, 1.39 to 1.72 mm, and 1.99 to 2.19 mm respectively.

As shown in Figure 3, the best image resolutions in the radial directions are at the CFOV of the PET system. The spatial resolution decreases dramatically along the radial offset within the initial 3 to 5 mm, and degrades much slower when the source shifts to the edge of the PET scanner. The spatial resolution of II coincidence is the best among all the three coincidences of II, IS, and SS. The spatial resolution of the IS coincidence is close to the spatial resolution of the II coincidence but is much lower than the spatial resolution of the SS coincidence.

### 3.2. System Sensitivity

**Figure 4:**
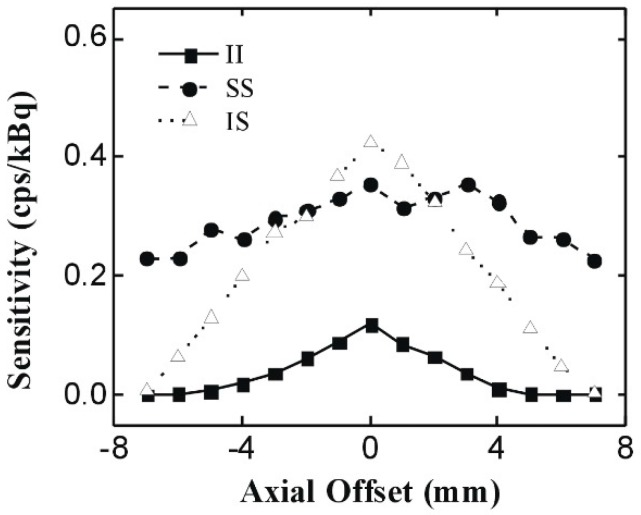
System sensitivity distribution in the axial directions, with an energy window of 350 to 650 keV PET imaging of point source and phantom.

Figure 4 shows the system sensitivity distribution in the axial directions, with an energy window of 350 to 650 keV. We selected the gamma-ray detection only within the insert ring range; in other words, from −7 to 7 mm. The entire width of the PET scanner ring was 23.4 mm along the axial direction, whereas the entire width of the insert ring was 9.6 mm. This made the SS coincidence range wider than the II and IS coincidence ranges.

As shown in Figure 4, the system sensitivity related to the IS coincidence shows a triangle profile. In the axial range from −7 to −2 mm, the sensitivity of the IS coincidence is lower than that of the SS coincidence but is higher than that of the II coincidence. In the range from −2 to 2 mm, the sensitivity of the IS became the highest among the three kinds of coincidences. The maximum sensitivity of the IS coincidence reaches 0.46 cps/kBq at the CFOV of the PET system, given the point source activity of 0.925 MBq. This phenomenon is explained by the higher gamma-ray detection efficiency of the CdZnTe detector and the shorter detection width of the insert detector. The sensitivity of the II coincidence approaches 0 at the edge of the PET scanner and gradually increases in the axial range from −7 to 0 mm. The maximum sensitivity of the II coincidence is 0.18 cps/kBq at the CFOV of the PET system. The sensitivity of the SS coincidence fluctuates significantly between 0.23 to 0.35 cps/kBq at the axial range of the insert ring. The sensitivity of the SS coincidence drops rapidly when the source is positioned toward the edge of the PET system.

In the CFOV of the system, the sensitivity of the IS coincidence is the highest, and the sensitivity of the II coincidence is the lowest. This tendency agrees well with the theory of VP-PET [1]. The spatial resolution and system sensitivity of our prototype PET indicates that the current design for a small-animal PET imaging system based on IS coincidence is reasonable and feasible.

### 3.3. PET Imaging of Point Source and Phantom

**Figure 5:**
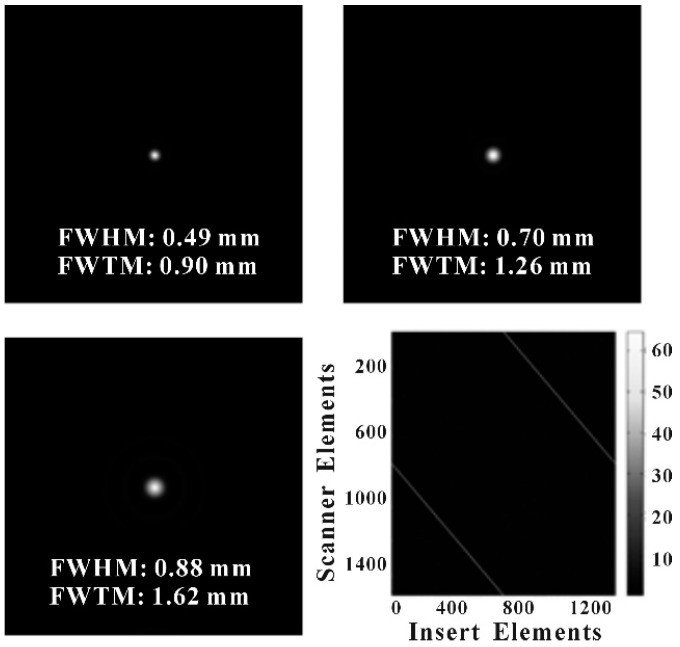
Reconstructed PET images of an ^18^F point source with a diameter of 0.5 mm for the insert–insert (II), insert-scanner (IS) and scanner-scanner (SS) coincidence events (from top to bottom). The gamma-ray detection matrix of the IS coincidence events is also shown in the bottom right of the figure.

Figure 5 shows the reconstructed image of an ^18^F point source with a diameter of 0.5 mm placed at the CFOV of the PET system. Three reconstructed images of the II, IS, and SS coincidence data are provided. The gamma-ray detection matrix for the IS coincidence is also shown in Figure 5 (bottom right), which is a separate straight line for a point source. The axis of the detection matrix corresponded to the crystal number of the insert ring and the PET scanner ring. Image resolutions at FWHM for the II, IS, and SS coincidence events were 0.49, 0.7, and 0.88 mm, respectively. The image resolution at FWTM for the II, IS, and SS coincidence events were 0.90, 1.26, and 1.62 mm, respectively. This result proves that the image resolution of the IS coincidence of VP-PET is better than the image resolution of the SS coincidence, which verifies that the inserted high-resolution detector improves the image resolution over that of the conventional PET system. The theoretical image resolution of the IS coincidence was 1.195 mm at FWHM. The GATE simulation results are in close agreement with the theoretical calculations when the positron range and acolinearity effects are included.

**Figure 6:**
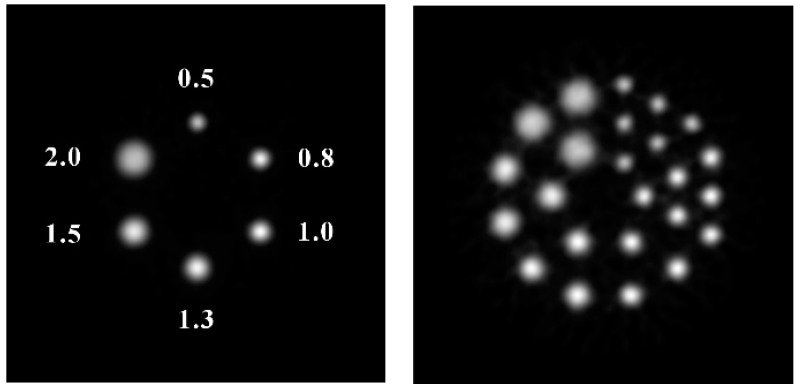
Reconstructed PET images of a rod source phantom and a Derenzo phantom for the IS coincidence events. The diameters of the sources from small to large were 0.5, 0.8, 1.0, 1.3, 1.5, and 2.0 mm.

Figure 6 shows the reconstructed image of a rod source phantom and a Derenzo phantom using IS coincidence data. The diameters of the six point sources were 0.5, 0.8, 1.0, 1.3, 1.5, and 2.0 mm. The distance from each source to the center was 8 mm, and the distance between two sources was also 8 mm. Note that all six point sources were clearly distinct. Figure 6 (right) shows the reconstructed image of a Derenzo phantom. The source dimension in the Derenzo phantom was the same as that in the rod source phantom. Figure 6 shows that the point sources of 0.5 mm in diameter can be clearly distinguished.

## 4. Discussion

**Figure 7:**
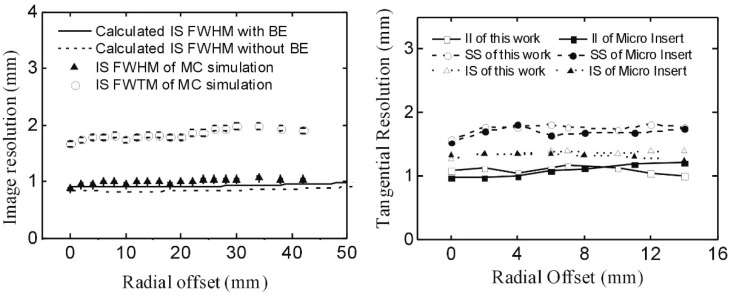
GATE-simulated image resolution of the current prototype PET system compared with the image resolution of the theoretical calculations (**left**) and the image resolution of the micro insert system [2] (**right**).

Virtual-pinhole PET (VP-PET) was originally defined and validated by researchers at Washington University in St. Louis (Tai and Wu et al.), and a submillimeter image resolution was obtained [1,2,3]. A study of a zoom-in PET was done by researchers at the University of California, Davis (Qi and Zhou et al.) [4,5]. In this article, we calculated the image resolution of our prototype system using the equations of VP-PET [1], and found very good agreement between the MC simulated results and the theoretical calculations, as shown in Figure 7 (left).

To compare the imaging resolution of the proposed PET system with that of the micro insert PET system designed at Washington University, we summarized the image resolution of the MC simulated results and the actual experimental results of the micro insert system, as shown in Figure 7 (right). The micro insert PET is the first VP-PET system [2] that integrated high-resolution LSO detectors into the MicroPET Focus-220. As we reported in a previous paper [6], the MC simulated results showed better image resolution than the real experimental results because (1) no electronic noise was included, and (2) no positron range and colinearity effect was simulated for back-to-back gamma-ray sources. In Figure 7 (right), the FWHM of the SS, IS, and II coincidences for the micro insert system was subtracted by a factor of 0.5, 0.4, and 0.2 mm, respectively, because the acolinearity of the PET system increased with the diameter of the PET scanner (SS > IS > II). After the correction, the image resolution of the proposed PET system was very consistent with the image resolution of the micro insert system.

For the imaging applications of highly pixelated CdZnTe detectors, the charge sharing effect was a challenge. We had demonstrated previously that if both double-pixel charge-sharing event and single-pixel photopeak event were included in the data acquisition process, the detection efficiency of the system would increase by 2.5 to 3 times [6]. The lack of charge sharing in this simulation would affect the positioning of events and the number of events detected. Due to the limitations of our MC codes, we did not include the charge sharing events in the GATE simulations. It should be investigated further for PET imaging applications of CdZnTe detector in both practical experiments and MC simulations.

## 5. Conclusions

In conclusion, a dedicated small-animal PET prototype system was simulated in GATE. The PET system had inner and outer radii of 129 and 315 mm, respectively, which composed a virtual pinhole structure to obtain high image resolution. The inner ring of the PET system consisted of 0.6 mm pixelated CdZnTe detectors, and the outer ring of the PET system comprised of 1.3 mm LYSO detectors. Our study shows that the system was able to achieve PET images with a resolution of 0.7 mm at FWHM using an FBP reconstruction algorithm. A GATE simulation suggested that the radial resolution of the reconstructed image was within 0.74 to 1.00 mm at FWHM, and the tangential resolution ranged from within 0.87 to 1.07 mm at FWHM. The system sensitivity at the CFOV of the PET system was 0.46 cps/kBq (with a source radioactivity of 0.925 MBq and an energy window of 350 keV to 650 keV). The GATE simulation indicated that sources with a diameter of 0.5mm could be clearly detected using 0.6 mm pixelated CdZnTe detectors as insert devices in a VP-PET system.
